# Lateral classification system predicts the collapse of JIC type C1 nontraumatic osteonecrosis of the femoral head: a retrospective study

**DOI:** 10.1186/s12891-023-06890-0

**Published:** 2023-09-26

**Authors:** Tianye Lin, Wensheng Zhang, Xiaoming He, Mincong He, Ziqi Li, Wei He, Zhenqiu Chen, Qingwen Zhang, Qiushi Wei

**Affiliations:** 1 Guangdong research institute for Orthopedics & Traumatology of Chinese Medicine, Guangzhou, 510405 Guangdong China; 2 Joint Center, The Third Affiliated Hospital of Guangzhou University of Chinese Medicine, Guangzhou, 510405 Guangdong China; 3 The First Affiliated Hospital of Guangzhou University of Chinese Medicine, Guangzhou, 510405 Guangdong China

**Keywords:** Osteonecrosis of femoral head, Lateral classification system, Midsagittal necrosis angle, Collapse

## Abstract

**Purposes:**

The aim of this study was to construct a lateral classification system for nontraumatic osteonecrosis of femoral head (NONFH) through three-dimensional reconstruction of the necrotic area to assist in evaluating the prognosis of patients with JIC type C1.

**Methods:**

Retrospective analysis of patients with JIC type C1 NONFH from January 2018 to December 2020. All patients were followed up for more than 3.5 years. The patients were divided into collapse group and non-collapse group according to whether the femoral head collapsed during the follow-up.Lateral classification system for femoral head necrosis is constructed through three-dimensional reconstruction of the necrotic area.Comparison of lateral classification system,midsagittal necrosis angle(MNA)and general data between the two groups.Furthermore, ROC curve analysis and survival analysis were performed.

**Results:**

318 patients were included in this study.There was a significant difference between the two groups in the lateral classification system (*P* < 0.05). In addition, the MNA in the collapsed group was significantly greater than that in the non-collapse group(*P* < 0.05). As revealed by the results of ROC analysis, the cutoff point of MNA was 104.5° (*P* < 0.05).According to the survivorship analysis, the mean survival time of the hips of patients with MNA less than 104.5°was greater than that of patients with MNA over 104.5° (*P* < 0.05). The survival rates of 3.5 years femoral head were 45.8%, 33.7%, 14.8%, 93.0%, and 100% for lateral classification system 1, 2, 3, 4, and 5, respectively.

**Conclusion:**

Necrosis involving the anterior aspect of the femoral head is an important risk factor for collapse. The Lateral classification system can effectively predict the femoral head collapse in JIC C1 type NONFH patients, supplementing the deficiency of JIC classification in evaluating the front of the femoral head.

## Introduction

Nontraumatic osteonecrosis of femoral head (NONFH) is a refractory orthopedic disease [1]. Due to a variety of non-traumatic reasons (hormone, alcoholism, etc.), the blood supply disorder of the femoral head causes osteocyte necrosis, which leads to the change of bone microstructure and abnormal bone metabolism in the femoral head, and finally leads to the decline of the bearing capacity of the femoral head [2]. If the intervention isn’t timely, the femoral head will collapse, and the affected hip will appear with joint pain, dysfunction, and other manifestations. In the pathological process of NONFH, femoral head collapse is a key issue in its treatment. The occurrence of femoral head collapse is the result of a combination of biology and biomechanics [3]. Collapse is the result of biomechanical abnormality of femoral head, the main reason being that osteonecrosis reduces the stress bearing capacity of unit trabecula. The pathological basis is a trabecular bone fracture, and its essence is “instability inner femoral head” [4, 5]. It is crucial to recognize this clinically because it defines the most basic treatment principle of NONFH. Therefore, early prediction of femoral head collapse in patients with NONFH has always been a research hotspot and difficulty.

At present, collapse assessment methods are mostly measured by staging classification methods. However, due to different generation times, factors such as the way of understanding the disease and the development of imaging technology lead to a different emphasis on each staging classification, and the accuracy of assessment is variable [6, 7]. The Japanese Investigation Committee (JIC) classification system, which classifies osteonecrosis of the femoral head based on the location of necrotic lesions, has been widely accepted and used throughout the world [8]. Because this classification is divided into acetabular weight-bearing areas, it is susceptible to femoral abduction, adduction changes, and whether there are congenital hip dysplasia and other diseases during measurement, thus affecting the evaluation results. Our previous study [9] followed 178 patients with osteonecrosis of the femoral head for 5 years and found that the collapse rate was 0% for type A hips, 24.3% for type B hips, 68.1% for type C1 hips, and 100% for type C2 hips. The results showed that type A and type B had slow progression and low collapse rate, and most type C2 would collapse, while which type C1 patients would collapse deserves our further study. The frog position of the hip joint is widely used to observe whether the extent of necrosis involves the anterior aspect, but it is affected by the shooting position and angle, particularly in patients with limited hip function [9]. Magnetic resonance imaging (MRI) or computed tomography (CT) scans are widely used to assess the extent and location of necrosis, however, it is difficult to reach a consensus on which layer should be taken to predict collapse [10, 11]. Therefore, an effective method of observing the anterior femoral head in NONFH patients is urgently needed.The three-dimensional reconstruction of the necrotic area can visually observe the three-dimensional distribution of the necrotic area, which is more conducive to judging whether the necrotic area involves the front of the femoral head. The purpose of this study is to construct a lateral classification system for femoral head necrosis through three-dimensional reconstruction of the necrotic area to assist in evaluating the prognosis of patients with JIC type C1. This study speculates that the lateral classification system of femoral head necrosis can effectively predict whether the femoral head collapses in patients with JIC C1 type and can provide early treatment options.

## Materials and methods

### Research objects

From January 2018 to December 2020, this study included patients with NONFH in our hospital.All patients included in the study met ONFH diagnosis [12]. Inclusion criteria were defined as (i) patients with JIC C1 ONFH in the anteroposterior view of the hip and no femoral head collapse at initial presentation, (ii) no history of hip trauma or surgery, and (iii) age between 18 and 55 years. Exclusion Criteria: Poor quality of existing imaging data (such as pelvic obliquity) at follow-up. Patients with cardiovascular or cerebrovascular disease, neurological disease, severe disease were also excluded. Demographic characteristics was recorded. They were divided into collapse and non-collapse groups according to whether the femoral head collapsed or not, and the criteria for femoral head collapse: crescent sign or visible collapse on follow-up radiographs [13]. This study has been approved by the Ethics Review Committee of the Third Affiliated Hospital of Guangzhou University of Traditional Chinese Medicine (No: PJ-KY-20220420-013).

### Midsagittal necrosis angle measurement

**Fig. 1:**
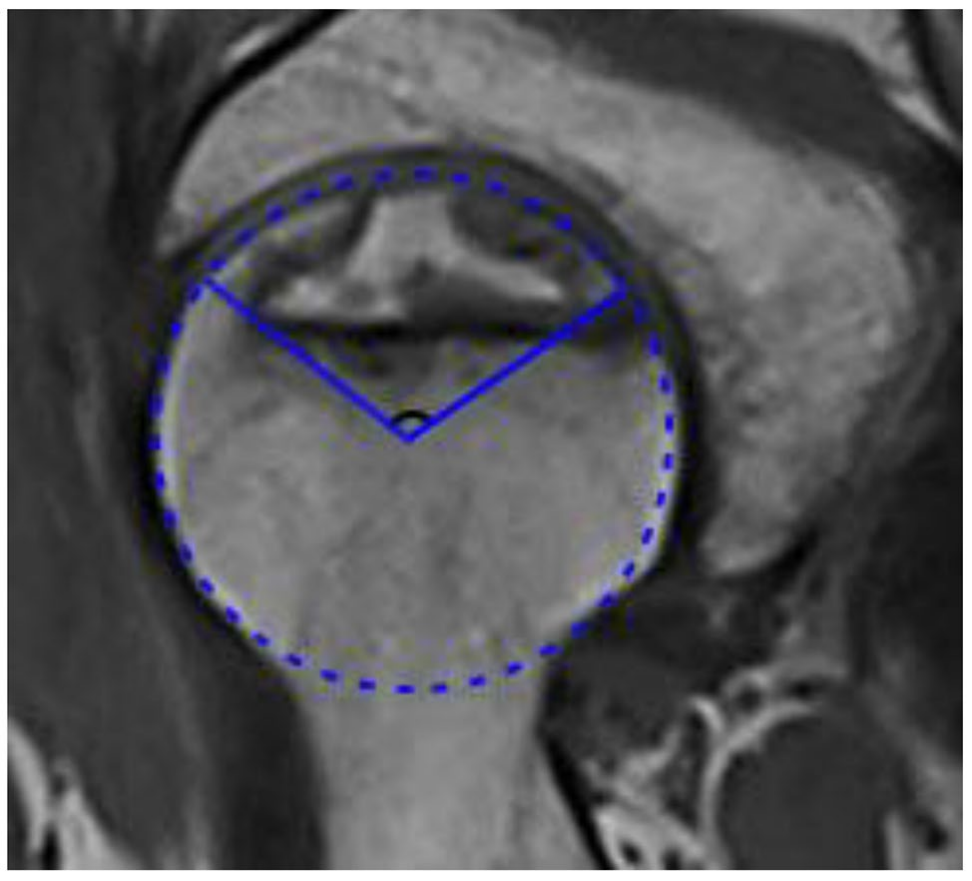
Midsagittal necrosis angle measurement

Referring to the previous research [14], MRI scans of patients were selected to measure the midsagittal necrosis angle(MNA): a tangent line was drawn on the midsagittal plane in front and behind the necrosis area, and the angle formed by the two tangent lines was the MNA(Fig. 1).

### Establish lateral classification system

**Fig. 2:**
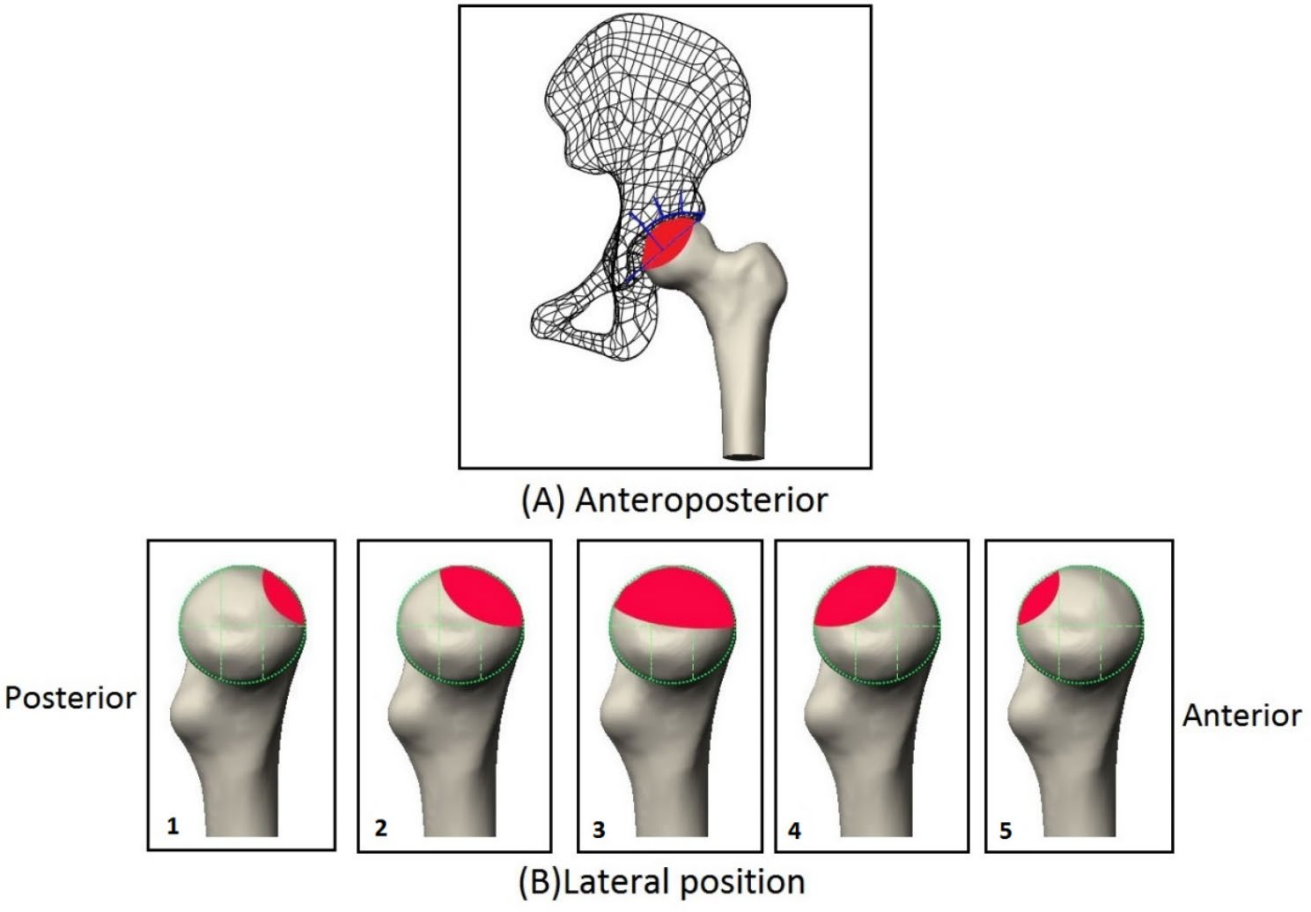
ONFH Lateral classification system

The proximal femur CT image data of a 40-year-old healthy male volunteer was selected and imported into Mimics 20.0 in DICOM format. The CT bone segmentation tool was used to create a calcaneus mask, and the mask was converted into a solid model and stored in STL format. On the side of the femoral head, divide the femoral head from front to back into three equal parts: front, middle, and back. Type 1: the necrosis range only involves the lateral front of the femoral head.Type 2: the necrosis range involves the front and middle of the femoral head. Type 3: the necrosis range involves the entire femoral head. Type 4: the necrosis range involves the middle and rear of the femoral head. Type 5:the extent of necrosis only involved the posterior part of the femoral head(Fig. 2).

### Evaluation of lateral classification system

**Fig. 3:**
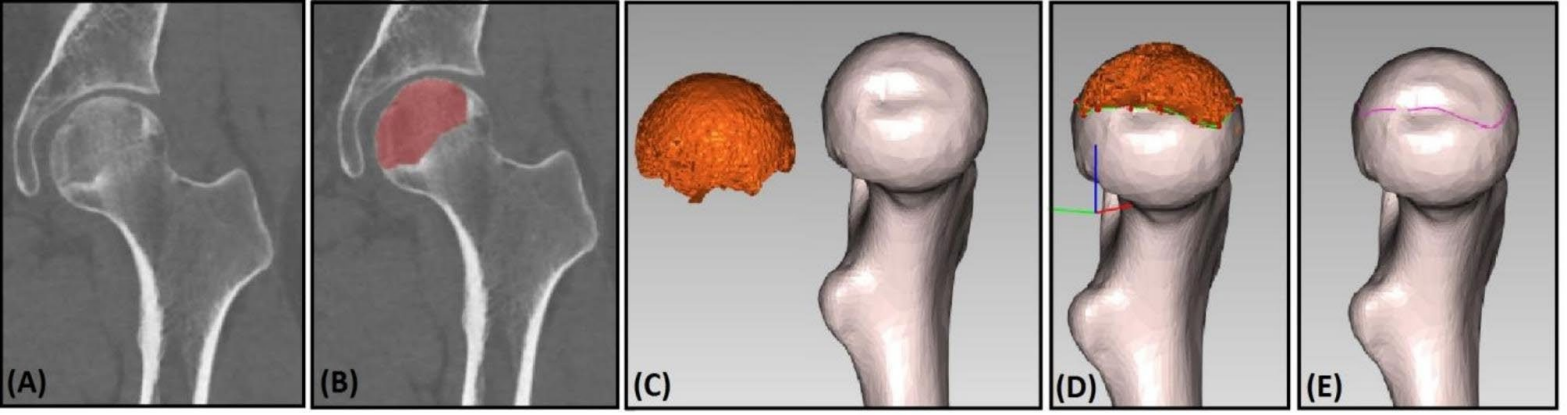
Create necrotic area outline and convert it to heat map (**A-C:** Construction of a standard model of femoral head and model of necrosis area. **D:** Matching of necrosis area with the standard model. **E:** Three-dimensional outline extraction of necrosis area)

Create the outline of the necrotic area and evaluate the lateral classification system: ①Import the above-mentioned standard proximal femur model into the E-3D software. ②The CT image data of patients with NONFH were followed by the same steps to create a necrotic area mask, and tools such as Edit Masks and Split Mask were used to separate the necrotic area, and the mask was converted into a solid model and imported into E-3D software in STL format. ③The femoral head standard template and necrosis area model was simultaneously imported through the E-3D software, and the standard model was matched with the necrosis area through the highest and lowest points of the fossa ovalis and the femoral head, and the contour line of the E-3D software was automatically extracted to obtain the contour line of the necrotic area in vitro is evaluated by the lateral classification system according to the contour(Fig. 3).

### Statistical methods

Statistical analyses were performed using SPSS 24.0 software (IBM Corp., Armonk, NY).Independent sample t-test was used to compare the results of the two groups. Chi-square test is used to analyze the results of classification data.The ROC curve was used to evaluate the cut-off point of MNA. Femoral head collapse was considered as the end point for Kaplan-Meier survival analysis. P values < 0.05 were considered statistically significant.

## Results

### Comparison of lateral classification system and general data between the two groups

Univariate analysis showed that there was a significant difference between the two groups in the lateral classification system (*P* < 0.05). Among them, the majority of the collapsed group was type 2 and 3,while the majority of the non-collapsed group was type 4. In addition, the MNA in the collapsed group was significantly greater than that in the non-collapse group, and the difference was statistically significant(*P* < 0.05). There was no significant difference between the two groups in terms of age, gender, etiology, etc. In addition, there was no significant difference in height, weight and BMI.(*P* > 0.05)(Table 1).

**Table 1:** Comparison of lateral classification system and general data between the two groups

Items	Non-collapse Group(n = 125(133 hips))	Collapse Group (n = 193(204 hips))	t /χ^2^	P value
Age (years)	40.74 ± 6.92	40.08 ± 7.63	0.772	0.441
Gender,n (%)				
Male	53(42.4%)	80(41.5%)	0.028	0.867
Female	72(57.6%)	113(58.5%)		
Height(cm)	165.54 ± 3.80	165.99 ± 5.76	-0.787	0.432
Weight(kg)	63.10 ± 8.77	62.79 ± 8.97	0.292	0.771
BMI(kg/m^2^)	23.07 ± 3.45	22.84 ± 3.40	0.584	0.560
Etiology,n(%)				
Steroid	56(44.8%)	75(38.9%)	1.226	0.542
Alcoholic	34(27.2%)	55(28.5%)		
Idiopathic	35(28.0%)	63(32.6%)		
Lateral classification system				
1	27(20.3%)	32(15.7%)	108.176	0.000
2	33(24.8%)	65(31.9%)		
3	18(13.5%)	104(51.0%)		
4	40(30.1%)	3(1.5%)		
5	15(11.3%)	0(0.0%)		
MNA	98.03 ± 16.12	115.05 ± 10.10	-9.121	0.000

### ROC analysis of MNA

**Fig. 4:**
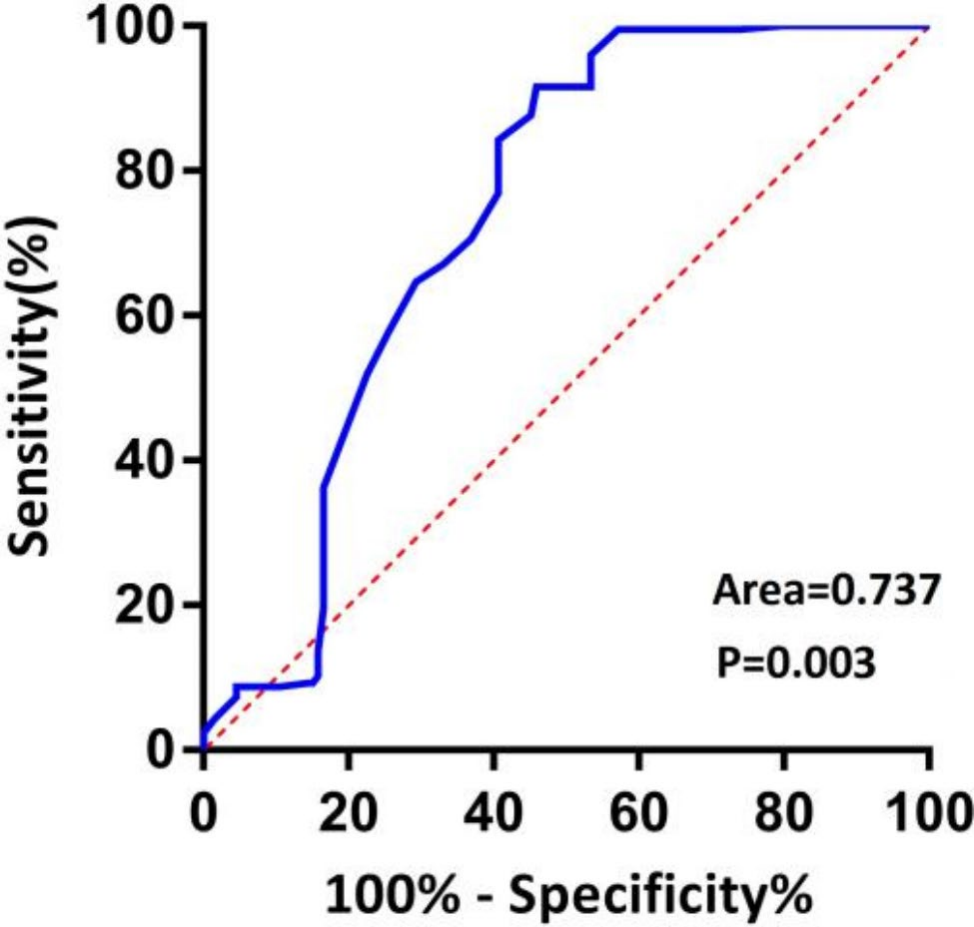
ROC curve analysis of MNA model for prediction of NONFH collapse compared with non-collapse NONFH

ROC analysis found that 104.5° was the cut-off point of MNA(sensitivity = 91.7%, specificity = 54.1%, *P* < 0.05).The area under the curve (AUC) values of MNA is 0.737 (Table 2 Fig. 4).

**Table 2:** Area under the ROC curve

Parameters	Area	Standard error	*P* value	Progressive 95% CI		Cutoff value	Youden’s index
				Lower limit	Upper limit		
MNA	0.737	0.031	0.003	0.676	0.798	104.5°	0.458

### Survivorship analysis of MNA and lateral classification system

**Fig. 5:**
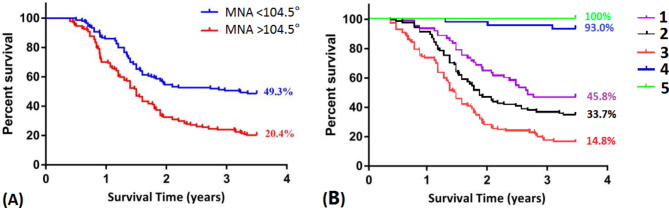
Survivorship analysis of MNA **(A)** and lateral classification system **(B)**

Collapse of the femoral head was used as the end point, the survival time of the hips of patients with small MNA (less than 104.5°) was significantly longer than that of patients with large MNA (more than 104.5°).The survival rate of femoral head at 3.5 years for hip with MNA < 104.5° was 51.7% (P < 0.05). (Fig. 5A). The survival rates of 3.5 years femoral head were 45.8%, 33.7%, 14.8%, 93.0%, and 100% for lateral classification system 1, 2, 3, 4, and 5, respectively(Fig. 5B).

## Discussions

Collapse of the femoral head is the most important pathological process of NONFH, and effective prediction of collapse is helpful to determine the prognosis of NONFH and provide treatment options. In order to find out which JIC C1 patients are more likely to have femoral head necrosis collapse, this study innovatively proposes the Lateral Classification system for femoral head necrosis. Patients were classified by the Lateral classification system based on the outline of the necrotic area by three-dimensional reconstruction of the necrotic area and registration in a standard femoral model, followed by extraction of the outline of the necrotic area edge. Low rates of collapse were found for types 4 and 5, and high rates for types 2 and 3. Lateral classification system is helpful to predict the collapse and evaluate the prognosis of NONFH JIC C1 patients.

Research suggests that early surgical intervention in NONFH can preserve the patient’s own hip joint as much as possible [15]. Early detection and timely treatment before femoral head collapse determine the prognosis of patients to a large extent, so the prediction and mechanism analysis of femoral head collapse has always been the main problem studied by domestic and foreign scholars. The location of necrotic lesions is closely related to the occurrence of femoral head collapse [16]. Necrotic lesions mostly occur in the upper, inner, and anterior regions of the avascular femoral head. It has been shown that lesions exceeding the medial two-thirds of the weight-bearing portion have a higher rate of collapse and faster stage progression, whereas femoral head collapse occurs more frequently when the lesion extends laterally to the acetabular rim [17]. It has been found that implanting a vascularized iliac bone flap into the anterolateral aspect of the necrotic femoral head is more effective [18]. Therefore, effective assessment of whether the necrotic area accumulates anterolateral superior femoral head is essential to determine collapse and prognosis in NONFH patients. JIC classification can easily and intuitively assess the degree of cumulative lateral wall in necrotic areas. In terms of assessing whether the necrotic area involves the lateral femoral head, the current staging and classification system is based on the imaging findings of the largest coronal level of the femoral head for analysis, and the imaging findings of the anterolateral femoral head are not considered [19]. Frog lateral radiographs were used to observe the anterolateral part of the femoral head and neck and were first used for the diagnosis of femorohip impingement [20]. Because attention is paid to the anterolateral column of the femoral head, frog lateral radiographs are gradually used as the main observation images for NONFH [21], but are affected by the patient’s position and filming angle. In addition, MRI midsagittal view has a role in assessing whether osteonecrosis of the femoral head involves the anterior aspect. Kubo et al. [22] predicted femoral head collapse by MRI sagittal anterior boundary necrosis angle and found that 27 patients with anterior femoral head necrosis angle less than 79° had only one femoral head collapse. Collapse occurred in all cases with anterior necrosis angles greater than or equal to 79°. However, the study was single center and the number of cases was limited. In this study, we investigated the relationship between MNA and femoral head collapse in NONFH patients and found that MNA in the collapse group was significantly greater than that in the non-collapse group, and further ROC curve analysis revealed that its determination value was 104.5°, which indicated that patients with MNA higher than 104.5° were prone to femoral head collapse. However, the AUC value of MNA is relatively low (0.737), therefore, the sensitivity and specificity of MNA for predicting femoral head collapse in NONFH patients still need to be further improved. MNA prediction inaccuracy may be due to the fact that the location of the anterior part of the necrosis assessed by two-dimensional images reflects the true location of irregular three-dimensional necrotic lesions, and the factors obtained by unilateral one section are compared with one-sided.

The location of necrotic lesions has an important impact on the progression of the disease, and studies suggest that femoral head necrosis located in weight-bearing areas is more likely to collapse [23]. Currently, there are octave methods [24] and latitude and longitude methods [25] for localization of necrotic sites, and such methods are complex and not particularly precise to use and are not accepted by a wide range of clinicians. In addition, there is a lack of effective methods to assess whether necrotic areas involve the anterior aspect of the femoral head. Three-dimensional reconstruction techniques are widely used in orthopedic clinical work, and volumetric measurements using MRI provide a more accurate assessment of lesion size in ONFH [26]. Mimics software can reconstruct the femoral head and necrotic lesions in three dimensions, display the shape, location and volume of osteonecrosis lesions in the femoral head stereoscopically, and then predict the progression of necrotic lesions and the occurrence of collapse. Hu et al. [16] found that three-dimensional reconstruction with Mimics software was able to accurately calculate the volume and percentage of necrosis compared with MRI and gross specimens. Zhao et al. [27] found through the finite element analysis (FEA) experiment that the probability of collapse of the femoral head is greater when the necrosis range is greater than 30%. When the necrotic volume is less than 30%, the necrotic site has a great influence on whether the femoral head collapses.In terms of treatment, Mishima et al. [28] successfully used 3D reconstruction technology to measure the lesion volume before and after conservative treatment of early necrotic femoral head, which is conducive to the formulation of preoperative surgical plan and the evaluation of the extent of sequestrum after surgery. Based on the three-dimensional reconstruction technology, this study reconstructed the necrotic area and further classified the three-dimensional position of the necrotic area. It was found that the collapse rate of type 4 and type 5 was low, while that of type 2 and type 3 was high. Type 4 and type 5 necrosis did not involve the front of the femoral head, while type 2 and type 3 involved the front of the femoral head with a large necrosis area, which may be the reason for the high collapse rate of type 2 and type 3. The location of the anterior border of the necrotic lesion plays an important role in the occurrence of collapse. Studies have shown that collapse may occur in ONFH patients with anterior necrosis even though the necrosis site is medial. The necrotic area of the patients selected in this study all involved the lateral femoral head (JIC C1 type). The lateral classification system is conducive to intuitively assessing whether the necrotic area involves the anterior side. The combination of the two is beneficial to the collapse prediction and prognosis evaluation of patients with NONFH JIC C1 type.Patients with Type 4 and type 5 have a lower rate of femoral head collapse and can be treated with non surgical hip preservation. For patients with type 1 and type 2, impact bone allocation and fiber grading can be used [29]. It is recommended to use hip relocation combined with bone graduation for patients with type 3 [30]. This is only the treatment plan provided for the Lateral Classification system based on our experience in treating NONFH, and the specific treatment effect still needs to be further verified through clinical studies on a large sample.

The innovation of this study is that the Lateral classification system was constructed for the first time and found to be effective in predicting the collapse of patients with JIC C1 type NONFH, supplementing the deficiency of JIC classification in evaluating the front of the femoral head. Despite the above research findings, there are still some limitations in this study: First, this study is a single-center study and patients with incomplete imaging data were excluded, which may have selection bias. Secondly, the mechanism of the lateral classification system to predict the collapse of the femoral head in patients with NONFH still needs further biomechanical experiments. Furthermore, the sizes of different necrotic femoral heads are not all the same, and when the necrotic area is matched to the standard proximal femoral model, there may be cases where it cannot be completely matched. But this situation has little impact on the classification image of the lateral classification system.

In conclusion, necrosis involving the anterior aspect of the femoral head is an important risk factor for collapse. The Lateral classification system can effectively predict the femoral head collapse in JIC C1 type NONFH patients, supplementing the deficiency of JIC classification in evaluating the front of the femoral head.

## Data Availability

The datasets generated during and analyzed during the current study are not publicly available due to datasets involves patient privacy but are available from the corresponding author on reasonable request.

## References

[1] Ma Hai-Yang, Ma Ning, Liu Yu-Feng. (2021) Core Decompression with Local Administration of Zoledronate and enriched bone marrow mononuclear cells for treatment of non-traumatic osteonecrosis of femoral Head. Orthop Surg, 13: p.1843–52. 10.1111/os.13100 PMC8523758 34664417

[2] Wu Zhong-Shu,Hong Guoju,Yang Peng (2020). The survival of non-traumatic osteonecrosis of femoral head at ARCO II with ring-shaped sclerotic zone: a mid-term follow-up retrospective study. J Hip Preserv Surg.

[3] Zheng Shao-Wei,Sun Chun-Han,Wen Zhi-Jia (2022). Decreased serum CXCL12/SDF-1 concentrations may reflect disease severity of non-traumatic osteonecrosis of femoral head. Clin Chim Acta.

[4] Chen W, Du Wei,Wu Panfeng (2022). Outcomes of free vascularized iliac bone flap for severe traumatic osteonecrosis of femoral head in young adults. Eur J Trauma Emerg Surg.

[5] Wang Q-RAJ-J. Zhang Wan-Li (2022) Core Decompression Prevents Progression of Asymptomatic Type C Osteonecrosis of Femoral Head According to the Japanese Investigation Committee Classification: A Retrospective Study. Orthop Surg, 14: p.851–859. 10.1111/os.13213 PMC9087457 35434904

[6] Ohzono K, Saito M, Takaoka K (1991). Natural history of nontraumatic avascular necrosis of the femoral head. J Bone Joint Surg Br.

[7] Steinberg ME, Hayken GD, Steinberg DR (1995). A quantitative system for staging avascular necrosis. J Bone Joint Surg Br.

[8] Sugano Nobuhiko, Atsumi Takashi, Ohzono Kenji (2002). The 2001 revised criteria for diagnosis, classification, and staging of idiopathic osteonecrosis of the femoral head. J Orthop Sci.

[9] Wei Q-S, He Min-Cong, He Xiao-Ming. (2022) Combining frog-leg lateral view may serve as a more sensitive X-ray position in monitoring collapse in osteonecrosis of the femoral head. J hip Preserv Surg, 9: p.10–7. 10.1093/jhps/hnac006 PMC9142202 35651706

[10] Han Xiaorui,Hong Guoju,Guo Yuan (2021). Novel MRI technique for the quantification of biochemical deterioration in steroid-induced osteonecrosis of femoral head: a prospective diagnostic trial. J Hip Preserv Surg.

[11] Liu Guang-Bo, Li Rui, Lu Qiang. (2020) Three-dimensional distribution of cystic lesions in osteonecrosis of the femoral head. J Orthop Translat, 22: p.109–15. 10.1016/j.jot.2019.10.010 PMC7231955 32440506

[12] Zhao DW, Hu YC (2012). Adult femoral head necrosis diagnosis and treatment standards expert consensus. Chin J Joint Surg (Electronic Ed.

[13] Kraus Meier Reinhard M, Schaeffeler Christoph. (2014) Bone marrow oedema on MR imaging indicates ARCO stage 3 disease in patients with AVN of the femoral head. Eur Radiol, 24: p. 2271–8. 10.1007/s00330-014-3216-8 24863885

[14] Ha Yong-Chan, Kim Hee Joong, Kim Shin-Yoon. (2010). Effects of age and body mass index on the results of transtrochanteric rotational osteotomy for femoral head osteonecrosis. J bone Joint Surg Am, 92: p.314 – 21. 10.2106/JBJS.H.01020 20124057

[15] Mont MA, Salem Hytham S, Piuzzi Nicolas S. (2020) Nontraumatic osteonecrosis of the femoral head: where do we stand today? A 5-Year Update. J Bone Joint Surg am, 102: p.1084–99. 10.2106/JBJS.19.01271 PMC7508290 32282421

[16] Hu LB, Huang ZG, Wei HY (2015). Osteonecrosis of the femoral head: using CT, MRI and gross specimen to characterize the location, shape and size of the lesion. Br J Radiol.

[17] Xin Pengfei, Tu Yonggang, Hong Zhinan. (2020) The clinical and radiographic characteristics of avascular necrosis after pediatric femoral neck fracture: a systematic review and retrospective study of 115 patients. J Orthop Surg Res, 15: p.520. 10.1186/s13018-020-02037-2 PMC7661253 33176837

[18] Nagoya S, Nagao M, Takada J (2004). Predictive factors for vascularized iliac bone graft for nontraumatic osteonecrosis of the femoral head. J Orthop Sci.

[19] Wu W, He W,Wei QS (2018). Prognostic analysis of different morphology of the necrotic-viable interface in osteonecrosis of. The Femoral head Int Orthop.

[20] Clohisy JC, Nunley RM,Otto RJ (2007). The frog-leg lateral radiograph accurately visualized hip cam impingement abnormalities. Clin Orthop Relat Res.

[21] Kang JS, Moon KH,Kwon DG (2013). The natural history of asymptomatic osteonecrosis of. The Femoral head Int Orthop.

[22] Kubo Y, Motomura G, Ikemura S (2018). The effect of the anterior boundary of necrotic lesion on the occurrence of collapse in osteonecrosis of the femoral head. Int Orthop.

[23] Sugano N, Ohzono K (2014). Natural course and the JIC classification of osteonecrosis of the femoral head. Osteonecrosis.

[24] Malizos K N, Siafakas M S, Fotiadis D I (2001). An MRI-based semiautomated volumetric quantification of hip osteonecrosis. Skeletal Radiol, 30: p.686 − 93. 10.1007/s002560100399 11810166

[25] Nishii T, Sugano N, Ohzono K (2002). Significance of lesion size and location in the prediction of collapse of osteonecrosis of the femoral head: a new three-dimensional quantification using magnetic resonance imaging. J Orthop Res.

[26] Hindoyan Kevork N, Lieberman Jay R, Matcuk George R (2020). A Precise and Reliable Method of determining lesion size in osteonecrosis of the femoral. Head Using Volumes J Arthroplasty.

[27] Zhao WPLF, Lu QP, Wu ZH (2005). 3D reconstruction and FEA in the prediction of osteonecrosis collapse of the femoral head. Chin J Biomed Eng.

[28] Mishima H, Sugaya H, Yoshioka T (2016). The safety and efficacy of combined autologous concentrated bone marrow grafting and low- intensity pulsed ultrasound in the treatment of osteonecrosis of the femoral head. Eur J Orthop Surg Traumatol.

[29] Chen L. Hong Guoju, Hong Zhinan (2020). Optimizing indications of impacting bone allograft transplantation in osteonecrosis of the femoral head.[J]. Bone Joint J, null: 838–844. 10.1302/0301-620X.102B7.BJJ-2019-1101.R2 32600141

[30] Chen W, Li Jianxiong, Guo, Wenxuan et al. (2022). Outcomes of surgical hip dislocation combined with bone graft for adolescents and younger adults with osteonecrosis of the femoral head: a case series and literature review.[J]. BMC Musculoskelet Disord, 23: 499. 10.1186/s12891-022-05456-w PMC9134689 35619082

